# Cost-effectiveness of the Perioperative Pain Management Bundle a registry-based study

**DOI:** 10.3389/fpubh.2023.1157484

**Published:** 2023-09-07

**Authors:** Suzana Bojic, Nebojsa Ladjevic, Ivan Palibrk, Ivan Soldatovic, Ivana Likic-Ladjevic, Winfried Meissner, Ruth Zaslansky, Ulrike M Stamer, Philipp Baumbach, Dusica Stamenkovic

**Affiliations:** ^1^Department of Anesthesiology and Intensive Care, University Hospital Medical Centre “Dr. Dragisa Misovic – Dedinje”, Belgrade, Serbia; ^2^Faculty of Medicine, University of Belgrade, Belgrade, Serbia; ^3^Centre of Anaesthesia and Resuscitation University Clinical Centre of Serbia, Belgrade, Serbia; ^4^Clinic for Gynecology and Obstetrics, University Clinical Centre of Serbia, Belgrade, Serbia; ^5^Department of Anesthesiology and Intensive Care, University Hospital Jena, Jena, Germany; ^6^Department of Anesthesiology and Pain Medicine, Inselspital, Bern University Hospital, University of Bern, Bern, Switzerland; ^7^Medical Faculty of Military Medical Academy, University of Defense, Belgrade, Serbia; ^8^Department of Anesthesiology and Intensive Care, Military Medical Academy, Belgrade, Serbia

**Keywords:** acute postoperative pain, Bundle, cost-effectiveness, cost-effectiveness plane, incremental cost-effectiveness ratio, international pain outcomes questionnaire, pain composite score, economic preference analysis

## Abstract

**Introduction:**

The Perioperative Pain Management Bundle was introduced in 10 Serbian PAIN OUT network hospitals to improve the quality of postoperative pain management. The Bundle consists of 4 elements: informing patients about postoperative pain treatment options; administering a full daily dose of 1–2 non-opioid analgesics; administering regional blocks and/or surgical wound infiltration; and assessing pain after surgery. In this study, we aimed to assess the cost-effectiveness of the Bundle during the initial 24 h after surgery.

**Materials and methods:**

The assessment of cost-effectiveness was carried out by comparing patients before and after Bundle implementation and by comparing patients who received all Bundle elements to those with no Bundle element. Costs of postoperative pain management included costs of the analgesic medications, costs of labor for administering these medications, and related disposable materials. A multidimensional Pain Composite Score (PCS), the effectiveness measurement, was obtained by averaging variables from the International Pain Outcomes questionnaire evaluating pain intensity, interference of pain with activities and emotions, and side effects of analgesic medications. The incremental cost-effectiveness ratio (ICER) was calculated as the incremental change in costs divided by the incremental change in PCS and plotted on the cost-effectiveness plane along with the economic preference analysis.

**Results:**

The ICER value calculated when comparing patients before and after Bundle implementation was 181.89 RSD (1.55 EUR) with plotted ICERs located in the northeast and southeast quadrants of the cost-effectiveness plane. However, when comparing patients with no Bundle elements and those with all four Bundle elements, the calculated ICER was −800.63 RSD (−6.82 EUR) with plotted ICERs located in the southeast quadrant of the cost-effectiveness plane. ICER values differ across surgical disciplines.

**Conclusion:**

The proposed perioperative pain management Bundle is cost-effective. The cost-effectiveness varies depending on the number of implemented Bundle elements and fluctuates across surgical disciplines.

## 1. Introduction

Estimated costs of surgical care account for up to 50% of all hospital expenditures (1). While acute postoperative pain management might not be as costly as some other aspects of surgical treatment, it must not be overlooked given its pivotal role in patients outcomes and healthcare system efficiency. Poorly controlled acute postoperative pain, reported in up to 80% of surgical patients, is associated with increased perioperative morbidity, prolonged opioid use, transition to chronic postoperative pain and, consequently, higher overall healthcare costs (2). Nevertheless, cost-effectiveness analyses of acute postoperative pain management strategies are scarce (3) and usually focused on particular perioperative pain management techniques and medications used after specific surgical procedures (4–16), making it difficult to generalize the findings.

In an effort to improve the efficacy of the acute perioperative pain management in Serbia, a Perioperative Pain Management Bundle was introduced (17). A “Bundle” is a small set of evidence-based interventions that, when implemented together in a defined patient population and care setting, result in significantly better outcomes than when implemented individually or not at all (18). Bundles have been employed to address a plethora of medical issues, including sepsis (19) and antimicrobial stewardship (20) but only recently acute postoperative pain (17). The aforementioned Perioperative Pain Management Bundle was established following the methodology outlined by PAIN OUT,^1^ an international research network and quality improvement registry focusing on perioperative pain management (21). The Bundle consists of 4 elements: (1) informing patients about postoperative pain treatment options, (2) administering a full daily dose of 1–2 non-opioid analgesics; (3) administering regional blocks and/or surgical wound infiltration; and (4) assessing pain after surgery. Implementation of the Bundle in a large cohort of patients undergoing diverse surgical procedures was associated with a significant reduction in pain-related patient-reported outcomes (PROs) (17). As a follow-up study, we wished to evaluate the cost-effectiveness of this approach. This is the first cost-effectiveness analysis of the Bundle used to advance acute perioperative pain management in patients facing various surgical procedures.

The most common method for evaluating the effectiveness of pain management in adults is through the use of a Numeric Rating Scale (NRS). This 11-point Likert scale is often employed to measure a single pain-related outcome such as pain intensity during periods of rest or movement (5, 6, 10–12, 22). However, postoperative pain is a multidimensional cognitive and emotional experience influenced by numerous factors, including but not limited to the perception of care and treatment-related side effects. Consequently, a comprehensive assessment of pain requires the use of multidimensional measurement tool. A recent innovative approach has emerged, integrating various pain-related PROs describing different aspects of pain into a Pain Composite Score (PCS) (17, 23–27). Cost-effectiveness studies focusing on acute postoperative pain management have yet to adopt the PCS as a metric for effectiveness.

In this study, we aimed to evaluate the cost-effectiveness of the Perioperative Pain Management Bundle. In the first step of the analysis, we compared patients treated before the Bundle implementation to patients treated after the Bundle implementation. In the second step, patients in whom all Bundle elements were applied were compared to those without any Bundle elements. The PCS was used as a measure of effectiveness. We hypothesize that the Bundle is a cost-effective strategy of improving the quality of acute postoperative pain management.

## 2. Materials and methods

### 2.1. Study design and setting

The PAIN OUT methodology for auditing perioperative pain on the first postoperative day (ClinicalTrials.gov, NCT02083835) was used to collect the data evaluated in the current study (21). The Serbian PAIN OUT network consisted of 10 government hospitals. All collaborators obtained permission from their local ethics committee to participate in the study.

Patients were enrolled if they fulfilled the following inclusion criteria: (1) were ≥18 years old; (2) were on the first postoperative day and returned to the ward from surgery for at least 6 h; (3) consented to take part in the study. Data from patients who underwent surgical procedures registered ≤10 times in the whole data set were excluded. For further analysis, surgical procedures were grouped according to similar expected pain levels (Tables 1, 2).

**Table 1:** Comparison of costs and Pain Composite Scores between Phase 1 (*before* the Bundle implementation) and Phase 2 (*after* the Bundle implementation) patients.

N (Phase 1/Phase 2)	Costs (RSD)		Pain Composite Score		ICER (RSD/USD)	
	Phase 1	Phase 2	Phase 1	Phase 2	RSD	EUR
Thyroidectomy (31/39)	324.41 (129.67–473.17)	685.41 (361.00–836.73)*	3.31 (1.46–4.15)	2.23 (1.46–2.77)	−334.26	−2.84
Breast surgery (148/176)	465.84 (232.92–698.76)	646.34 (413.42–736.83)*	1.46 (0.77–3.130)	1.77 (1.08–2.77)	582.26	4.96
Cardiac surgery (95/85)	1474.68 (1040.01–1755.61)	2916.75 (2389.07–3241.16)*	1.92 (1.25–2.75)	1.38 (0.77–1.92)*	−2670.37	−22.76
Major abdominal surgery (301/156)	1262.50 (707.02–2129.96)	1450.70 (1091.43–2125.65)*	2.75 (1.85–4.00)	1.77 (1.08–2.80)*	−192.04	−1.64
Laparoscopic cholecystectomy (104/102)	778.80 (465.84–1244.64)	785.01 (96.72–1124.69)	2.40 (1.32–3.85)	1.88 (1.23–2.54)*	−11.92	−0.10
Appendectomy (8/8)	1264.97 (933.48–1420.66)	643.17 (385.27–774.67)*	2.50 (1.65–4.31)	2.46 (1.42–3.92)	15542.50	132.41
Inguinal hernia repair (97/79)	1244.64 (595.51–12852.13)	969.37 (361.00–12548.09)	1.92 (0.92–2.92)	1.85 (1.23–2.85)	3931.43	33.49
Nephrectomy (46/47)	1978.43 (1933.43–2510.74)	2510.74 (1933.43–2530.83)	2.36 (1.31–3.15)	2.23 (1.54–2.77)	−4094.62	−34.88
Other urology (48/45)	1933.43 (1933.43–2206.42)	2510.74 (1933.43–3050.84)*	1.31 (0.68–2.24)	1.69 (1.00–2.33)	1519.21	12.91
Cesarean section (31/66)	698.76 (563.74–1439.07)	736.83 (556.33–945.73)	3.38 (2.62–5.00)	3.77 (2.08–4.67)	97.53	0.83
Other gynecology (13/18)	1336.13 (1168.20–1477.56)	942.32 (608.75–1361.43)	2.62 (1.31–4.46)	3.00 (2.38–4.31)	−1009.74	−8.60
Fracture fixation (55/27)	741.96 (368.41–12566.79)	829.32 (512.32–1673.47)	2.18 (1.23–3.38)	2.17 (0.92–2.85)	−8740.00	−74.46
Hip/knee replacement (188/203)	2655.11 (714.40–13722.42)	1933.43 (988.49–13720.68)	1.84 (1.17–3.28)	2.00 (1.31–2.75)	−4510.63	−38.43
All (1,165/1,051)	1193.81 (547.11–2141.43)	1124.69 (647.58–2462.92)*	2.23 (1.25–3.50)	1.85 (1.23–2.85)*	181.89	1.55

Data are presented as frequencies and median (25–75th percentile). ICER, Incremental Cost-Effectiveness Ratio; RSD, Republic of Serbia Dinar; EUR, Euro. *Statistically significant difference between patients, Mann Whitney U test, *p* < 0.05.

**Table 2:** Comparison of costs and Pain Composite Scores between patients with no Bundle elements and patients with all Bundle elements.

N (No Bundle/Full Bundle)	Costs (RSD)		Pain Composite Score		ICER	
	No Bundle elements	All Bundle elements	No Bundle elements	All Bundle elements	RSD	EUR
Thyroidectomy (11/29)	291.88 (129.67–324.41)	692.82 (368.41–836.73)*	3.31 (1.46–5.31)	2.23 (1.31–2.77)	−371.24	−3.16
Breast surgery (39/24)	465.84 (232.92–698.76)	700.24 (375.83–836.73)*	1.69 (0.92–3.54)	1.12 (0.85–2.04)	−411.22	−3.50
Cardiac surgery (28/31)	1126.01 (618.37–1535.71)	3104.67 (2592.34–3429.08)*	2.25 (1.42–3.04)	1.08 (0.62–2.08)*	−1691.16	−14.41
Major abdominal surgery (20/84)	547.11 (393.17–910.06)	1552.88 (1091.43–2192.79)*	2.64 (0.96–3.72)	1.50 (1.08–2.29)	−882.25	−7.52
Laparoscopic cholecystectomy (57/30)	791.61 (648.82–1244.64)	949.68 (310.17–1153.73)	2.38 (1.15–3.85)	1.85 (1.23–2.92)	−298.25	−2.54
Appendectomy (4/2)	1180.21 (933.48–1420.66)	547.15 (394.72–699.57)	2.50 (1.81–4.62)	3.19 (0.77–5.62)	−917.48	−7.81
Inguinal hernia repair (30/30)	778.80 (465.84–1206.15)	951.59 (361.00–12548.09)	2.35 (0.54–2.92)	1.65 (1.23–2.69)	−246.84	−2.10
Nephrectomy (9/7)	2080.02 (2023.44–2404.43)	2510.74 (2510.74–2510.74)*	2.58 (1.54–2.85)	1.85 (1.69–3.15)	−590.03	−5.03
Other urology (10/2)	1933.43 (1557.60–2206.42)	8291.16 (1175.52–15406.80)	2.19 (1.77–3.08)	1.85 (1.15–2.54)	−18699.20	−159.30
Cesarean section (10/16)	698.76 (698.76–804.41)	575.25 (527.21–753.69)	4.08 (2.69–5.00)	3.44 (1.88–4.19)	192.98	1.64
Other gynecology (6/3)	1219.22 (960.89–1381.72)	959.30 (925.33–1154.63)	3.12 (1.31–5.92)	3.15 (2.62–5.62)	−86640.00	−738.11
Fracture fixation (14/19)	1011.72 (465.84–2141.43)	700.24 (512.32–1349.06)	1.24 (0.85–1.62)	1.92 (0.92–2.67)	−458.06	−3.90
Hip/knee replacement (33/106)	1634.04 (420.83–2130.46)	1198.53 (724.99–13178.02)	2.00 (1.42–2.83)	2.08 (1.38–2.92)	−5443.88	−46.38
All (271/383)	778.80 (465.84–1401.12)	1147.09 (700.24–2510.74)*	2.23 (1.25–3.50)	1.77 (1.08–2.75)*	−800.63	−6.82

Data are presented as frequencies and median (25–75th percentile). ICER, Incremental Cost-Effectiveness Ratio; RSD, Republic of Serbia Dinar; EUR, Euro. *Statistically significant difference between patients with no Bundle elements and all Bundle elements, Mann Whitney U test, *p* < 0.05.

In each participating hospital, trained surveyors obtained the study data during the first 24 h after surgery. In Phase 1 (January–May 2018), baseline data were collected before the introduction of the Bundle. In Phase 2 (April–December 2019), the Bundle was introduced, and another set of data was acquired.

### 2.2. Assessment of costs

Costs of postoperative pain management included: (1) costs of the analgesic medications, (2) costs of staff labor needed for administering the analgesic medications, and (3) costs of related disposable materials. Costs of hospitalization and staff fees were considered fixed.

The costs of the analgesic medications were calculated by multiplying the number of vials of each medication used in the recovery room and ward with the unitary price of the vial. The number of vials used was derived from the cumulative dose of the medication, as recorded per PAIN OUT protocol, divided by the dose of that medication in the vial. If the medication was available in vials containing various doses, the price of the vial containing the larger dose was used. If the medication was administered in a dose smaller than that in a vial, the price for the whole vial was utilized.

The costs of labor needed for administering the analgesic medications were calculated as follows. For the intravenous, intramuscular, or subcutaneous route of administration, the costs of labor were calculated by multiplying the number of used vials with the price of labor for the intravenous, intramuscular, or subcutaneous administration of medications. A unitary price was adopted for costing of neuraxial or peripheral nerve block administration.

Costs of disposable materials related to analgesic therapy were estimated assuming “ideal consumption” whereby disposable materials are used without waste.

All prices were obtained from the official price list of the Republic of Serbia National Health Insurance Fund.^2^ The prices of medications, labor, and disposables are equal across all government hospitals in Serbia.

### 2.3. Assessment of effectiveness

Per PAIN OUT protocol, pain-related PROs were obtained from patients using the International Pain Outcomes questionnaire (IPO-Q). The IPO-Q asks patients to evaluate their postoperative pain in terms of its *intensity* (worst and least pain, time spent in severe pain), *the extent it interferes* with activities (in bed, with coughing), sleep and emotions (anxiety and feeling of helplessness), *side effects* of analgesic medications (nausea, drowsiness, itchiness and dizziness), and how they *perceive the care* they receive, on an 11-point Numerical Rating Likert scales (28, 29).

The multidimensional PCS was calculated as an average of the continuous variables in the *IPO-Q’s pain intensity, interference* and *side effects* domains (29). Higher PCS values indicate worse outcomes.

### 2.4. Assessment of cost-effectiveness

The cost-effectiveness of the Perioperative Pain Management Bundle was assessed using the incremental cost-effectiveness ratio (ICER). The ICER was calculated as the incremental change in median costs divided by the incremental change in the median PCS (30). It will, therefore, be negative if median costs increase but median effectiveness decreases, and vice versa. If both median costs and effectiveness increase or decrease, the ICER will have a positive value.

In the first step of our analysis, we calculated ICER by dividing the difference in median costs of postoperative pain management by the difference in median PCS between Phase 2 and Phase 1 of the PAIN OUT study (Equation 1). With this approach, we evaluated the Bundle cost-effectiveness independent of the number of Bundle elements that individual patients received.


(1) $$\begin{array}{c} \mathrm{ICER}=\mathrm{Median cost of postoperative analgesia}\;\left(\mathrm{Phase}\;2-\mathrm{Phase}\;1\right)\\ /\mathrm{Median}\;\mathrm{PCS}\;\left(\mathrm{Phase}\;2-\mathrm{Phase}\;1\right). \end{array}$$

In the second step of the cost-effectiveness analysis, we compared patients in whom all four Bundle elements were applied with those in whom no Bundle elements were applied (Equation 2).


(2) $$\begin{array}{c} \mathrm{ICER}=\mathrm{Median cost of postoperative analgesia}\;\\ \left(\mathrm{Full Bundle}-\mathrm{No}\;\mathrm{Bundle}\right)/\mathrm{Median}\;\mathrm{PCS}\\ \;\left(\mathrm{Full Bundle}-\mathrm{No}\;\mathrm{Bundle}\right) \end{array}$$.

The cost-effectiveness was also presented graphically on the cost-effectiveness plane using the method described by Bang and Zhao (30) along with the economic preference analysis developed by Obenchain (31). In the cost-effectiveness plane, cost increments were plotted on the Y-axis so that more negative values were unambiguously more favorable. The effectiveness increments were plotted on the X-axis to make the larger, more positive values unambiguously more favorable. Therefore, the northwest quadrant of the plane represents highly unfavorable situations where a cost increase is coupled with a decrease in effectiveness. The opposite, highly favorable situations where an increase in effectiveness is coupled with a decrease in costs are positioned in the southeast quadrant. In the northeast quadrant, the intervention increases both costs and effectiveness, and the opposite stands for the southwest quadrant (32, 33). Results of the economic preference analysis are displayed as colors of the spectrum, with the red end of the spectrum as the least favorable and the green end of the spectrum as the most favorable (31).

Costs and, consequently, ICERs were calculated in Republic of Serbia Dinars (RSD) and then converted to Euros (EUR) according to the middle exchange rate published by the National Bank of Serbia on the day the price list was downloaded (September 16th, 2022).

### 2.5. Statistical analysis

Data were analyzed using SPSS 20.0 (IBM SPSS Statistics for Windows, Armonk, NY) and R 3.4.2 (R Core Team, 2017, Vienna, Austria). Results are presented as count and median with 25th – 75th percentile or 95% Confidence intervals. Groups of patients were compared using the Mann–Whitney *U* test. The ICEinfer package in R for Windows was used to obtain graphical representation of the cost-effectiveness analysis. This package makes head-to-head treatment comparisons by generating bivariate bootstrap resampling distribution of ICE for a specified value of the shadow price of health and lambda, forms ICE confidence region with specified confidence fraction, color bootstrap outcomes within confidence wedge from ICE map with specified values of lambda, beta and gamma. Parameters lambda = 0.26, gamma = 1 and beta = 1 were used in the calculations (30, 31). *p* values less than 0.05 were considered significant.

## 3. Results

The cost-effectiveness analysis was performed on 2,216 patients after excluding data from 138 patients who underwent infrequent surgical procedures.

### 3.1. Cost-effectiveness when comparing Phase 1 and Phase 2 of the PAIN OUT project

When evaluating the complete cohort, costs of postoperative pain management were significantly higher and PCS lower in Phase 2 compared to Phase 1 patients. However, the resulting ICER was positive since the median costs of postoperative pain management in Phase 2 were lower than in Phase 1 (Table 1).

Costs were significantly higher in Phase 2 patients undergoing thyroidectomy, breast, cardiac and major abdominal surgery, and urological procedures other than nephrectomy. However, only in patients who underwent cardiac and major abdominal surgery costs increased in parallel with a significant decrease in the PCS, resulting in negative ICER values. In patients with thyroidectomy and breast surgery, higher costs of postoperative pain management in Phase 2, together with similar values of PCS between phases, were observed. Costs did not differ between the two project phases for laparoscopic cholecystectomy patients, but the PCS values were significantly reduced in Phase 2. Interestingly, in patients undergoing appendectomy, costs were lower in Phase 2, with no difference in PCS between the phases. There were no significant changes in costs and PCS between Phase 1 and Phase 2 for patients undergoing inguinal hernia repair, nephrectomy and other urological procedures, Cesarean section and other gynecological procedures, fracture fixation and hip/knee replacement (Table 1).

**Figure 1:**
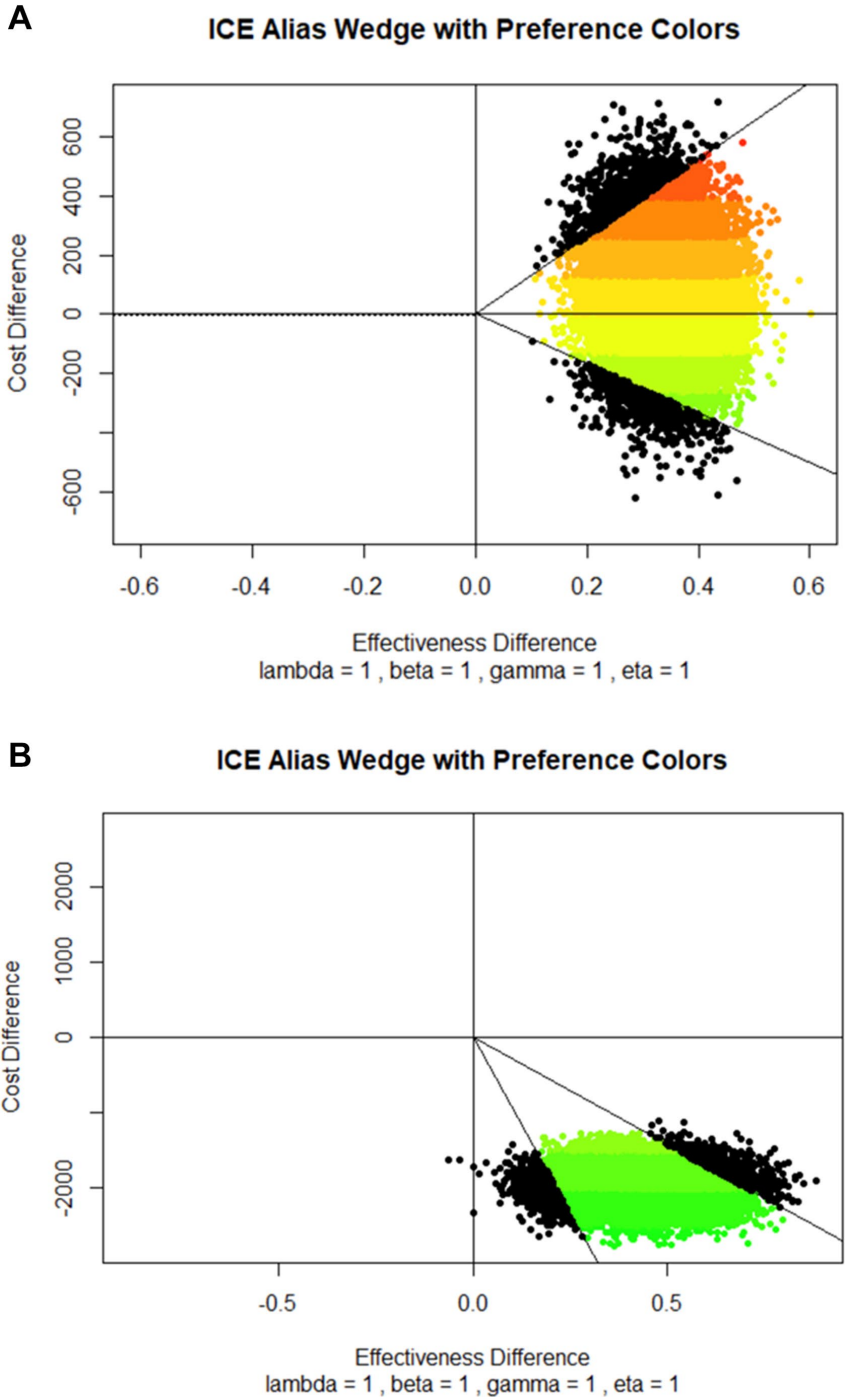
Cost-effectiveness planes with economic preference colors based on the comparison between **(A)** Phase 1 (before the Bundle implementation) and Phase 2 (after the Bundle implementation) patients, and **(B)** patients with no Bundle elements and patients with all Bundle elements. Cost increments were plotted on the Y-axis so that more negative values were more favorable. The effectiveness increments were plotted on the X-axis to make the more positive values more favorable. Full lines represent 95% C.I. Results of the economic preference analysis are displayed as colors of the spectrum, with the red end of the spectrum as the least favorable and the green end of the spectrum as the most favorable.

Plotted ICERs are positioned in the northeast and southeast quadrants of the cost-effectiveness plane (Figure 1A), consistent with the positive and negative ICER values calculated for different surgical procedures and disciplines (Table 1).

### 3.2. Cost-effectiveness when comparing patients with no Bundle elements and all Bundle elements

Results of the cost-effectiveness analysis that compared patients with no Bundle elements to those receiving all four Bundle elements are presented in Table 2. The costs of postoperative pain management in 383 (17.28%) patients in whom all four elements of the Bundle were applied were significantly higher, and PCS significantly lower than in 271 (12.23%) patients in whom no Bundle elements were applied. The resulting calculated ICER was negative with a higher absolute value than ICER calculated when comparing Phase 1 and Phase 2 patients.

Implementing all four Bundle elements resulted in significantly higher costs of postoperative pain management in patients undergoing thyroidectomy, breast, cardiac and major abdominal surgery, and nephrectomy. However, PCS scores were lower only in cardiac surgery patients (Table 2).

Plotted ICERs were almost entirely concentrated in the southeast quadrant of the cost-effectiveness plane (Figure 1B), suggesting that the Bundle is more effective and saves money than the no-Bundle approach.

## 4. Discussion

Our results show that the proposed four-element Perioperative Pain Management Bundle is cost-effective and that the cost-effectiveness varies depending on the number of implemented Bundle elements and across surgical disciplines.

The Perioperative Pain Management Bundle described in our study is the first example of using a Bundle approach to improve the efficacy of acute postoperative pain management across multiple surgical disciplines. Earlier cost-effectiveness studies in acute postoperative pain focused on individual surgical procedures or analgesic medications, making the results challenging to extrapolate to other settings. McDowell et al. (7) assessed the cost-effectiveness of different ketamine infusion protocols after Chiari I decompression. The cost-effectiveness of liposomal bupivacaine in multimodal postoperative pain management strategies after total knee replacement and major abdominal surgery gave conflicting results (4–6). Intravenous acetaminophen with or without ketorolac reduced the cost of care compared with opioids-alone following scoliosis surgery in adolescents (8). Wound infiltration was economically beneficial in patients undergoing cardiac surgery (9), as was transcranial direct current stimulation after thoracotomy (10). Costs of treating adverse events were lower when post-surgical pain was treated with oliceridine compared to morphine (14). Tapentadol IR was more cost-effective than oxycodone IR after major hip surgery (15).

Evaluating the costs of perioperative pain management is a highly complex task. Unlike the prices of medications and disposables, which are usually readily available and, therefore, easily comparable in different economic settings, the price of labor varies significantly not only between developed and developing countries (13) but also within each country. In the current study, all hospitals were government-founded; thus, the prices of labor, medications and disposables are equal in all hospitals and determined by the government.

However, the results of the cost-effectiveness analysis might be affected by parameters not addressed in this paper such as medications used for treating side-effects of anesthesia and analgesic medications, comorbidities, duration of hospital stay or selection of pain medications administered to patients. This study was performed using registry data so introduction of new variables is not feasible. The lack of information about the costs of medications for treating medication-related adverse events is the limitation of our study, given that these costs can influence the cost-effectiveness of acute postoperative pain management (14). Our results focus on pain management offered to patients during the first 24 h after surgery. Pain scores during this period are unlikely to be affected by the duration of the hospital stay. PAIN OUT registry contains data on multiple comorbidities that might affect postoperative pain management. There was no significant difference in prevalence of comorbidities between patients with all four Bundle elements (76.2%) and those who did not receive all Bundle elements (74.5%). Also, the availability of certain pain medications in Serbia is likely different than in other countries which might limit generalizability of the findings to other countries. However, the aim of the study is to demonstrate that the principle of good care can be economically favorable and not to calculate the exact reduction or increase in costs.

This is the first cost-effectiveness analysis in the field of acute postoperative pain that uses PCS as a measure of effectiveness. We calculated PCS as an average value of 12 IPO-Q variables since there is no evidence that one variable or a set of variables describes the overall experience of pain more closely than other variables. In recent studies, however, PCS was calculated as a weighted score, with some variables contributing more than others (23, 25, 27).

ICER is generally accepted in pharmacoeconomics as the preferred analytical tool for cost-effectiveness analysis. It is usually calculated using mean values of costs and effectiveness outcome. However, when dealing with skewed data, such as in this study, a median is considered a more appropriate measure of central tendency. The difference in ICER values derived from the same dataset using mean and median values can often be very different, impacting statistical inference (30).

This difference in ICER values and the location of plotted ICERs in two steps of the analysis may be explained by the implementation of all four Bundle elements only achieved in 17.28% patients. In contrast, other patients received one, two or three Bundle elements. Therefore, the cost-effectiveness of the Bundle may depend on the number of Bundle elements implemented, and our findings indicate that implementing all four elements is the most favorable. However, the continuation of this analysis would infer a “threshold ICER” defined by the amount that society is willing to pay to treat acute postoperative pain. Serbian National Health Insurance Fund has yet to define this sum. Willingness-to-pay sum defined by patients (34, 35) depends on the local and individual socioeconomic situation and is, therefore, difficult to extrapolate to different settings.

Our results show that ICER values vary across surgical disciplines suggesting different cost-effectiveness of the Bundle after different surgical procedures. A possible explanation could be in procedure-specific differences in postoperative pain intensity (36) and the unequal distribution of the more costly analgesic techniques in different surgical disciplines. For example, costly epidural analgesia is likely used in major abdominal surgery but not for thyroidectomy, where systemic analgesics and/or cervical plexus blocks, which are less costly, would be more appropriate.

As discussed above, future cost-effectiveness evaluation of the Perioperative Pain Management Bundle should include data on analgesic medication-related adverse events and compare alternate PCS calculations. We propose to investigate the Bundle in surgical procedures not included here, in patients with countries and settings with different socioeconomic backgrounds and referring to data collected during the entire hospital stay of a patient.

## 5. Conclusion

This study evaluated the cost-effectiveness of the four-element Perioperative Pain Management Bundle. Our results suggest that the implementation of the Bundle is a cost-effective strategy for improving the quality of perioperative pain management.

## Data availability statement

The raw data supporting the conclusions of this article will be made available by the authors, without undue reservation.

## Ethics statement

The studies involving humans were approved by (1) Uzice Health Center, General hospital Prijepolje - Ethical approval for this study (Ethical Committee N° 0303/7804) was provided by the Ethical Committee of Uzice Health Center, Uzice, Serbia (Chairperson Dr. Sladjana Pavic) on 13 July 2017. (2) Cardiovascular Institute Dedinje - Ethical approval (Ethical Committee N° 4301 dated Sept 21, 2017) was provided by the Ethical Committee of Institute for Cardiovascular diseases Dedinje, Belgrade, Serbia (Chairperson Prof. Dr. Dragan Sagic) on 21 Sept 2017. (3) Oncology Institute of Vojvodina - Ethical approval for this study (Ethical Committee N° 4/17/1-2204/2-6) was provided by the Ethical Committee of Oncology Institute of Vojvodina, Sremska Kamenica, Serbia (Chairperson Prof S. Knežević Ušaj) on 17 July 2017. (4) Department of Anesthesia, Urology Clinic, Faculty of Medicine, University of Belgrade - Ethical approval (Ethical Committee N° 361/14-A) was provided by the Ethical Committee of Clinical Centre of Serbia, Belgrade, Serbia (Chairperson Prof. Dr. Branislav Stefanovic) on 13 July 2017. [*] (5) Military Medical Academy Department of Anesthesiology and Intensive Care - Ethical approval for this study (Ethical Committee Military Medical Academy MMA TS/24.10.2017) was provided by the Ethical Committee Military Medical Academy, Belgrade, Serbia (Chairperson Colonel Prof. Nebojsa Jovic, Deputy Chairperson Prof. Viktorija Dragojevic-Simic) on 3 November 2017. (6) Clinical Hospital Centre Bezanijska kosa- Belgrade - Ethical approval for this study (Ethical Committee of Clinical Hospital Centre Bezanijska Kosa N°7622/3) was provided by the Ethical Committee Clinical Hospital Centre Bezanijska Kosa (Chairperson Dr. Mirjana Cvetkovic) on 8 September 2017. (7) Clinical Centre Nis - Ethical approval for this study (Ethical Committee Protocol number: 27771/12) was provided by Ethical Committee of the University of Nis, Clinical Centre Nis, Serbia (Chairperson: Dr. Steva Stanisic) on 5 September 2017. (8) National Cancer Research Center of Serbia, Belgrade - Ethical approval for this study (Ethical Committee No 5246-01) was provided by the Ethical Committee of the National Cancer Research Center of Serbia (Chairperson Dr. Bosnjak, MD, PhD) on November 14th, 2017. (9) Centre for Anesthesiology, Resuscitation and Intensive Care. University Clinical Centre of Serbia; Department of Anesthesia, Clinic for Digestive Surgery - Ethical approval for this study (Ethical Committee No 361/14-B) was provided by the Ethical Committee of University Clinical Center of Serbia, Belgrade, Pasterova 2, Serbia (Chairperson Prof B Stefanović) on 13 July 2017. (10) Clinical Centre of Serbia, Clinic for Physical and Rehabilitation Medicine, Faculty of Medicine, University of Belgrade - Ethical approval (Ethical Committee N° 361/14-A) was provided by the Ethical Committee of Clinical Centre of Serbia, Belgrade, Serbia (Chairperson Prof. Dr. Branislav Stefanovic) on 13 July 2017. [*]. The studies were conducted in accordance with the local legislation and institutional requirements. Written informed consent for participation was not required from the participants or the participants’ legal guardians/next of kin in accordance with the national legislation and institutional requirements.

## Author contributions

SB and DS: conception and design. SB, DS, NL, IP, and IL-L: acquisition of the data. SB and IS: analysis or interpretation of data. SB, DS, and RZ: drafting of the article. IS and PB: statistical analysis. SB, NL, IP, IS, IL-L, WM, RZ, US, PB, and DS: critical revision of the manuscript for important intellectual content. All authors contributed to the article and approved the submitted version.

## Funding

PAIN OUT was developed with funding from the European Community’s Seventh Framework Program FP7/2007-2013 under Grant Agreement No. 223590. The European Pain Federation (EFIC) provided funding from its own resources to cover the costs of the project in Serbia. Funding included: (i) the annual subscription to PAIN OUT for 10 hospitals over a 2-year period; (ii) two half-day face-to-face meetings so that the Principal Investigator and one research surveyor from each hospital could review the findings and (iii) partial remuneration to hospitals for datasets collected. The funds were transferred to the Serbian Pain Association, which then contacted each of the participating hospitals. Neither funding body received or requested the trial protocol, analysis plan, analysis itself or any drafts of the manuscript prior to submission for publication.

## Conflict of interest

The authors declare that the research was conducted in the absence of any commercial or financial relationships that could be construed as a potential conflict of interest.

## Publisher’s note

All claims expressed in this article are solely those of the authors and do not necessarily represent those of their affiliated organizations, or those of the publisher, the editors and the reviewers. Any product that may be evaluated in this article, or claim that may be made by its manufacturer, is not guaranteed or endorsed by the publisher.

## Footnotes

^1^ www.pain-out.eu

^2^ https://www.rfzo.rs
